# Towards the creation of decellularized organ constructs using irreversible electroporation and active mechanical perfusion

**DOI:** 10.1186/1475-925X-9-83

**Published:** 2010-12-10

**Authors:** Michael B Sano, Robert E Neal, Paulo A Garcia, David Gerber, John Robertson, Rafael V Davalos

**Affiliations:** 1 School of Biomedical Engineering and Sciences, Virginia Tech - Wake Forest University, Blacksburg, VA, USA; 2 Department of Surgery, School of Medicine, University of North Carolina, Chapel Hill, NC, USA; 3 Department of Biomedical Sciences & Pathobiology, Virginia-Maryland Regional College of Veterinary Medicine, Blacksburg, VA, USA; 4 Wake Forest Institute of Regenerative Medicine, Wake Forest University, Winston-Salem, NC, USA

## Abstract

**Background:**

Despite advances in transplant surgery and general medicine, the number of patients awaiting transplant organs continues to grow, while the supply of organs does not. This work outlines a method of organ decellularization using non-thermal irreversible electroporation (N-TIRE) which, in combination with reseeding, may help supplement the supply of organs for transplant.

**Methods:**

In our study, brief but intense electric pulses were applied to porcine livers while under active low temperature cardio-emulation perfusion. Histological analysis and lesion measurements were used to determine the effects of the pulses in decellularizing the livers as a first step towards the development of extracellular scaffolds that may be used with stem cell reseeding. A dynamic conductivity numerical model was developed to simulate the treatment parameters used and determine an irreversible electroporation threshold.

**Results:**

Ninety-nine individual 1000 V/cm 100-μs square pulses with repetition rates between 0.25 and 4 Hz were found to produce a lesion within 24 hours post-treatment. The livers maintained intact bile ducts and vascular structures while demonstrating hepatocytic cord disruption and cell delamination from cord basal laminae after 24 hours of perfusion. A numerical model found an electric field threshold of 423 V/cm under specific experimental conditions, which may be used in the future to plan treatments for the decellularization of entire organs. Analysis of the pulse repetition rate shows that the largest treated area and the lowest interstitial density score was achieved for a pulse frequency of 1 Hz. After 24 hours of perfusion, a maximum density score reduction of 58.5 percent had been achieved.

**Conclusions:**

This method is the first effort towards creating decellularized tissue scaffolds that could be used for organ transplantation using N-TIRE. In addition, it provides a versatile platform to study the effects of pulse parameters such as pulse length, repetition rate, and field strength on whole organ structures.

## Background

Over the past fifty years, organ transplantation has become a standard care for patients diagnosed with end stage organ failure including cirrhosis and renal failure. Liver transplantation is very successful, with 90 and 75% survival rates after 1 and 5 years, respectively. Unfortunately, the number of patients with cirrhosis, chronic viral hepatitis and hepatocellular carcinoma has steadily increased, leading to unmet demands for organ transplantation [1]. According to the United Network of Organ Sharing (UNOS), there are over 108,000 candidates in the US alone currently waiting for organ transplants including kidney, liver, heart, and lung. In 2009, there were fewer than 7,000 liver transplants from both living and deceased donors [2].

Despite advances in transplant surgery and general medicine, the number of patients awaiting transplant organs continues to grow, while organ supply does not. Organ supply is constrained by obstacles that impede acquisition, such as the requirement for organ removal coincident with brainstem death necessitating the use of hospital resources to maintain artificial life support. As a result, organ donation may be problematic when intensive care resources are strained[3]. In addition, life support for potential organ donations has been ethically debated[4,5] and donation refusal is common in regions where social, cultural, and religious pressures constrain organ procurement.

The increasing gap between organ donation and supply to severely-ill patients has fostered an increased interest in alternative organ sources[6]. For the development and differentiation of full organs suitable for human transplant, structures that provide microvasculature for the delivery of nutrients to all cells must be developed[7-9]. Traditional top-down manufacturing techniques are currently unable to produce a hierarchical vascular structure scale which can span the more than 4 orders of magnitude of human organs[10]. Microfabrication techniques can replicate some features of the complex architecture of mammalian microvasculature, but current processes fail to extend into the macro-scale[11]. Thus, structures which have features spanning multiple length scales are currently only fabricated through biological mechanisms and the relatively new field of biofabrication has developed, with the goal of utilizing and manipulating these processes [12].

Decellularization of existing tissues extends the concept of biofabrication by taking advantage of the body's natural programming to create a complete tissue, including a functional vascular network. Rat liver extracellular matrix constructs have been created using chemical decellularization and reseeding [13-15]. Decellularized rat hearts, reseeded with multiple cell types, can contract and have the ability to generate pumping pressures [16]. Challenges to chemical decellularization techniques include the potential for detergents to damage extracellular matrix components [17,18] the potential to create and deposit toxins [13,17], and the inherent difficulty of scaling these techniques up from small rat organs to larger organs [14]. These challenges must be overcome before decellularized organs can successfully be translated to the clinical setting.

Xenotransplantation, or the transplantation of animal organs, is one potential solution to the future organ shortages [19]. Porcine xenotransplants have shown considerable potential but have failed to become widely accepted or used clinically. Transplantation of porcine pancreatic islets has recently been shown to temporarily reverse diabetes mellitus [20,21] and the use of T-cell tolerance protocols have demonstrated feasibility of long-term renal xeonograft transplantation in a non-human primate model [22]. Additionally, it has been shown that explanted porcine livers have the ability to clear ammonium and restore coagulation while under short term perfusion of human plasma [23,24]. Unfortunately, the mechanisms of graft loss and rejection in these transplants are still not well understood, and immunological rejection remains a significant barrier to successful transplantation [25].

Hypothermic oxygenated perfusion (HOPE) is a method of whole organ preservation which mechanically delivers an oxygenated, nutrient-rich blood substitute to an entire organ at sub-physiological temperatures [26,27]. This method has been successfully demonstrated to improve the preservation quality and transplant success rates of kidneys which have undergone warm ischemia [28,29]; with research striving to reach 72 hour preservation times [30]. In addition, Schon *et al. *[31] and Brockmann *et al. *[32] have demonstrated the ability to prolong organ quality using normothermic perfusion, a process in which the perfused fluid is held at or near physiological temperatures. These methods of organ preservation can be used to isolate N-TIRE tissue ablation effects from the immune response observed *in vivo *and the natural degradation of tissue post mortem.

Electroporation is a non-linear biophysical process in which the application of pulsed electric fields leads to an increase in permeability of cells, presumably through the creation of nanoscale pores in the lipid bilayer [33]. At low pulsing energy, this permeability is reversible and cellular health and function is maintained. Once a critical electric field intensity threshold is surpassed (approximately 500 [34] to 700 V/cm [35] for ninety 50 *μ*s pulses at 4 Hz in brain and eight 100 μs pulses at 1 Hz in liver, respectively), the cell membrane is unable to recover and cell death is induced in a precise and controllable manner with sub-millimeter resolution [36,37]. This process is referred to as non-thermal irreversible electroporation (N-TIRE) [38]. N-TIRE does not rely on thermal mechanisms [38] and preserves the structure of the underlying extracellular matrix as well as nerve conduits and bile ducts [39]. Since N-TIRE cell death does not require any drugs, there should not be any creation or deposition of toxins when killing the cells from this technique.

Recently, we and others have determined, through the use of translational laboratory models, that capitalizing on the ability of N-TIRE to destroy cells without destroying the extracellular matrix might make N-TIRE a viable means for scaffold creation via organ decellularization [40,41]. We hypothesize that viable decellularized tissue scaffolds can be obtained using non-thermal irreversible electroporation (N-TIRE) on organs under continuous perfusion.

Machine-perfused porcine livers were treated with N-TIRE using external plate or needle electrodes within one hour of organ harvest and establishment of active perfusion. At varying time points after electroporation, livers were removed from perfusion, immediately after which samples were collected, preserved in 10% neutral buffered formalin, prepared for histology, and their microscopic structure was examined. Examination of the N-TIRE treated and control (untreated) regions of tissue demonstrated that N-TIRE was capable of decellularizing large volumes of tissue when performed in conjunction with active organ perfusion, suggesting that N-TIRE may be a viable method of decellularization for tissue engineering applications.

## Methods

### Tissue

Young mixed breed pigs were sacrificed via barbiturate overdose. Livers were harvested and placed on ice within 15 minutes of death. Vascular anastomosis with the perfusion system was created by inserting Luer lock syringe connections into the portal vein, hepatic artery, and major hepatic vein and then secured with zip ties. The livers were flushed with lactated Ringer's solution (LRS) to remove blood/clots before placement on the perfusion system.

### Perfusion

The VasoWave™ Perfusion System (Smart Perfusion, Denver, NC) was used to perfuse the livers for 4 and 24 hours. This system produces a cardioemulating pulse wave to generate physiological systolic and diastolic pressures and flow rates within the organ. The system is capable of controlling the oxygen content of the perfusate above and below physiological norms. A perfusate, consisting of modified LRS, was delivered to the portal vein and hepatic artery and recycled back into the system via the hepatic vein. All livers were under active machine perfusion within one hour post-mortem and the perfusate was held at 4°C

### Electroporation

**Figure 1:**
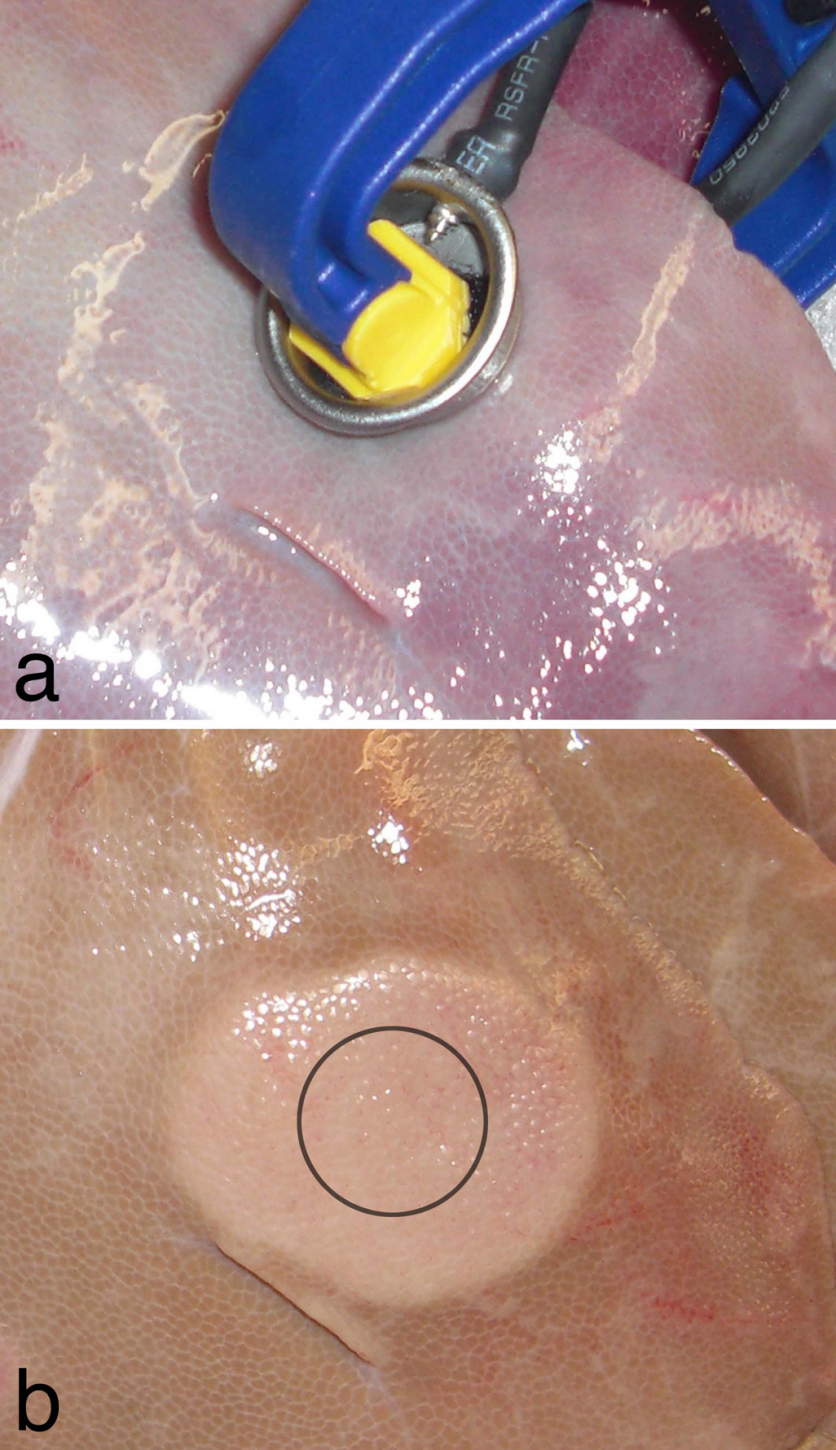
**Experimental setup and IRE lesion**. (a) Placement of the electrodes on actively perfused liver tissue and (b) the resultant lesion after treatment with 99, 100 μs, 1500 V/cm pulses and 4 hours of perfusion. The approximate area of the electrode is outlined in black.

The ECM 830 Square Wave BTX Electroporation System (Harvard Apparatus, Cambridge, MA) was used to deliver low-energy pulses to the liver tissue while it was on ice undergoing active perfusion with the solution maintained at 21°C. Two metal plate electrodes, 2 cm in diameter, were attached to a pair of ratcheting vice grips (38 mm, Irwin Quick-Grips) using Velcro. High voltage wire was used to connect the electrodes to the BTX unit. The electrodes were clamped gently to the liver and the center-to-center distance between the electrodes was measured. The voltage output on the BTX unit was adjusted such that the approximate applied electric field was 1000 V/cm. Then, ninety-nine individual 100-μs square pulses were administered at repetition rates of 0.25, 0.5, 1.0 and 4.0 Hz. Repetition rates trials were performed at random and repeated a minimum of three times. Sham controls were performed by placing the electrodes over the tissue without delivering any pulses. Since needle electrodes are typically employed in clinical applications of IRE, two additional trials were performed using needle electrodes separated by 0.5 cm, inserted into the tissue approximately 1 cm, using a voltage-to-distance ratio of 1500 V/cm at rates of 1 and 4 Hz. The experimental setup for plate electrodes is illustrated in Figure 1a. All N-TIRE treatments were completed within two hours post mortem. The surface lesion created at each treatment site was measured at the end of the 24 hour perfusion period. Statistical analysis of the lesion diameters was conducted using JMP 8.0 (SAS Institute Inc., South Cary, NC) via Student's t-test with a 0.1 *α *level. Histological images were imported into ImageJ (Version 1.43u, NIH, USA). For each sample, a binary image was created using the threshold tool based on a sample selected within an acellular region. An average pixel value for each image was calculated using the measure RGB plugin and a density score was created by normalizing these values to 1; where 1 corresponds to regions filled with cells and extracellular material and 0 to a region completely devoid of material. Samples were analyzed for statistical significance in JMP via Student's t-test with a maximum 0.05 *α *level and a minimum of 6 samples for each treatment group.

### Tissue preparation

Following N-TIRE treatment and machine perfusion, livers were disconnected from the VasoWave™ system, immediately sectioned to preserve lesions, and tissues were immediately fixed by immersion in 10% neutral buffered formalin solution. After fixation, tissues were trimmed and processed for routine paraffin embedding, then sectioned at 4 micrometers, and stained with hematoxylin-eosin (H&E) or Masson's trichrome stain. Tissue sections were evaluated by a veterinary pathologist who had no knowledge of the N-TIRE treatment parameters.

### Numerical model

**Figure 2:**
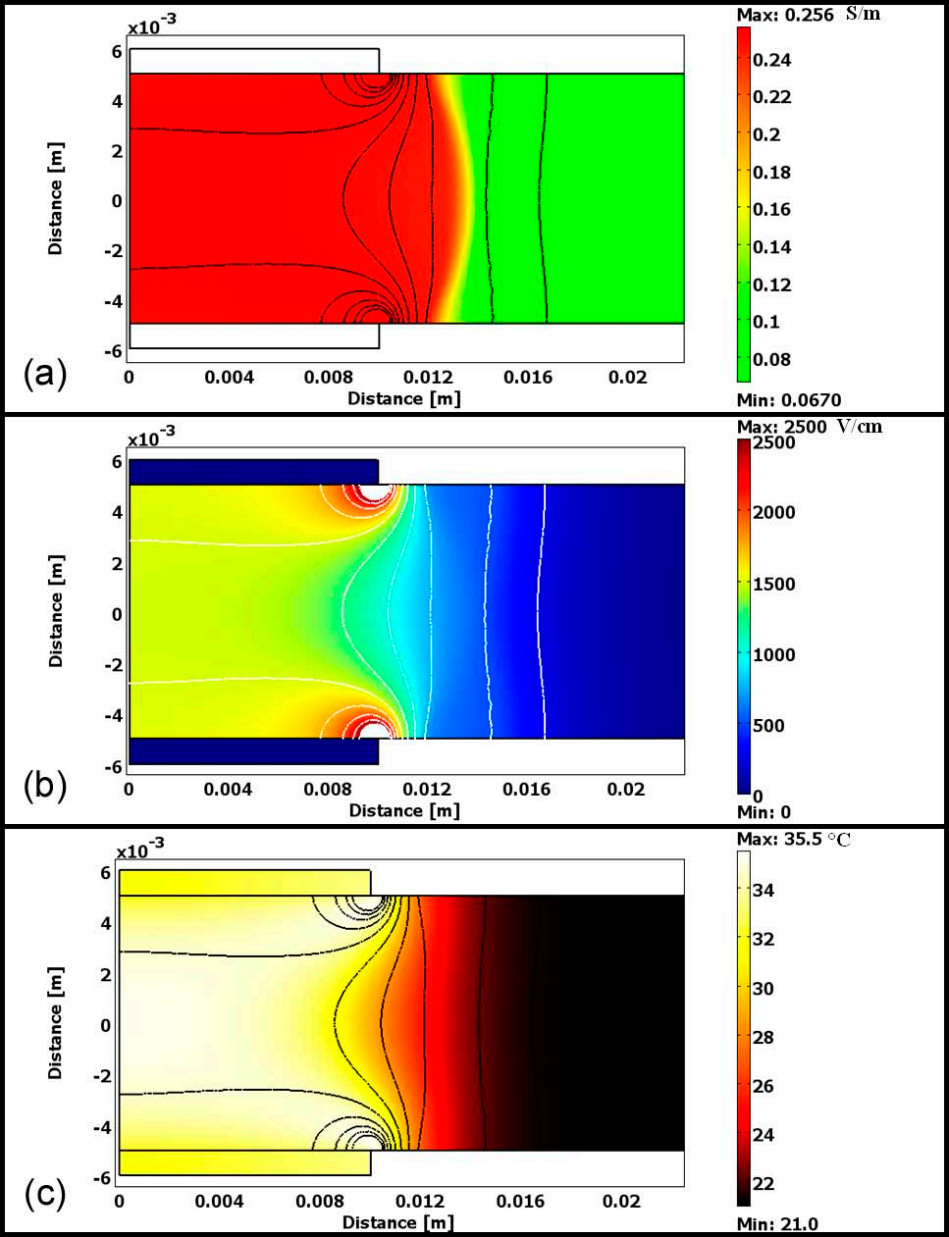
**Numerical model of the liver tissue with conductivity map, electric field distribution, and thermal map**. (a) The conductivity of the tissue changes from 0.067 to 0.256 during treatment based on changes in the (b) electric field distribution and (c) temperature changes.

Numerical modeling can be used to predict the electric field distribution, and thus provide insight into the N-TIRE treatment regions in tissue [42,43]. This has been chosen as the method to correlate lesion volume with electric field in the liver. The methods for predicting N-TIRE areas are similar to the ones described by Sel *et al. *[35]. In order to understand the effective electric field threshold to induce N-TIRE in the liver, finite element simulations were conducted using Comsol Multiphysics 3.5a (Comsol, Stockholm, Sweden). The numerical model was constructed using 2 cm diameter plates, each 1 mm thick, placed above and below the tissue. The model was generated in an axis symmetric platform and the conductivity changes incorporated the effects of electroporation and temperature as described by Garcia *et al. *[34], with identical parameters from [35] and its physical setup may be seen in Figure 2. The electric field distribution is given by solving the Laplace equation:

∇⋅(σ∇ϕ)=0

where σ is the electric conductivity of the tissue and φ is the potential. The electrical boundary condition along the tissue that is in contact with the energized electrode is φ = V_o_. The electrical boundary condition at the interface of the other electrode is φ = 0.

The boundaries where the analyzed domain is not in contact with an electrode are treated as electrical insulation.

Conductivity changes due to electroporation and temperature have been modeled to calculate the dynamic conductivity according to the following equation:

$$\begin{array}{c} \sigma_{dynamic}\left(normE\_dc\right)\\ =\sigma_{0}\left[1+flc2hs\left(normE_{dc}-E_{delta},E_{range}\right)+\alpha \left(T-T_{o}\right)\right] \end{array}$$

where *σ*_0 _is the baseline conductivity. *flc*2*hs *is a smoothed heavyside function with a continuous second derivative that ensures convergence of the numerical solution. This function is defined in Comsol, and it changes from zero to one when *normE_dc *- *E_delta _*= 0 over the range *E_range_*. However, any continuous step function may be used to model the conductivity change, depending on the application, such as the sigmoidal ones proposed in [35,44]. In the *flc2hs *function that mimics the sigmoidal ones, *normE*_*dc *is the magnitude of the electric field, and *E_delta _*is the magnitude of the electric field at which the transition occurs over the range, *E_range_*. In the simulations, we used *E_delta _*= 580 V/cm and *E_range _*= ±120 V/cm. These values were selected from the literature in which models incorporated conductivity changes due to electroporation and were validated with real-time measurements in rat and rabbit liver [35,45]. The baseline tissue conductivity was set to 0.067 S/m [35], and N-TIRE affected tissue was considered to increase by a factor of 3.6 as determined by Sel *et al. *[35,46], reaching a final conductivity of 0.241 S/m. The electric field within the tissue domain was first determined using a conductivity of 0.067 S/m, adjusted to incorporate the dynamic conductivity, and reevaluated to determine the final electric field distribution. This numerical model was solved for the pulse parameters that produced the maximum thermal effects (i.e. 1500 V/cm at 4 Hz) used on the livers in order to obtain a simulation of the electric field to which the tissue was exposed using the 1.5% °C^-1 ^(Δσ/σ/ΔT) temperature coefficient in electrical conductivity. The temperature was calculated with the Penne's bioheat equation with the additional joule heating term [47] and with the values of the liver tissue heat capacity (c_p _= 3.6 kJ·kg^-1^K^-1^), thermal conductivity (k = 0.512 W·m^-1^K^-1^), density (ρ = 1050 kg·m^-3^), blood perfusion per unit volume (w_b _= 1 kg·m^-3^s^-1^), and the heat capacity of blood (c_p _= (3.64 kJ·kg^-1^K^-1^) taken from the literature [48,49]. The outer surface of the analyzed liver domain and top electrode surface was mathematically considered to have proportional loss to air due to convective heat transfer, h = 10W/(m^2 ^· K), as in [50] with *T*_∞ _= 21°C. The electrode-tissue boundaries were treated as continuity. The N-TIRE electric field thresholds were then found by measuring lesion dimensions and determining the electric field value at this region in the model after the completion of the 99 pulses and thus incorporating all the thermal effects as well.

## Results

Surface lesions develop during perfusion within 30 minutes initiating of treatment. The area of these on the liver surface created by plate electrodes were larger than, but the same type as that from the needle electrodes. In Figure 1b, a 3.3 cm surface lesion produced from an applied voltage of 1500 V may be seen, taken 4 hours after treatment. Numerically modeled, this lesion size was produced within the region of tissue experiencing an electric field of 423 +/- 147 V/cm (average ± standard deviation). The results of the numerical model for this trial may be seen in Figure 2.

**Figure 3:**
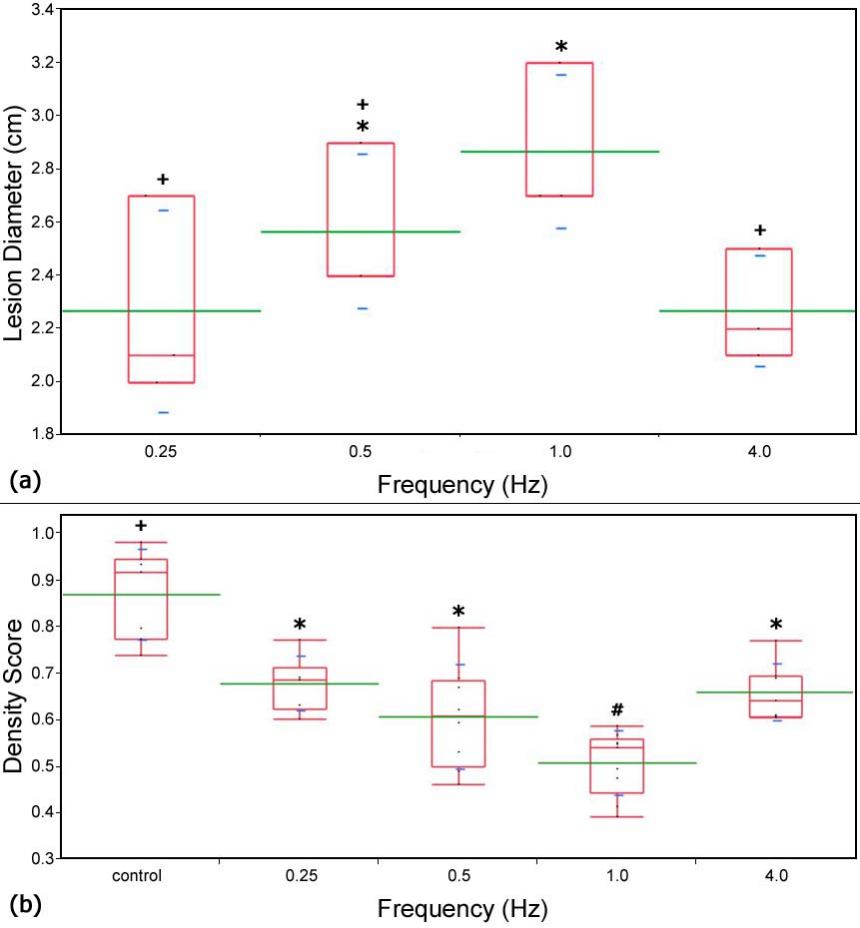
**Lesion diameter and density score vs. pulse frequency**. Plots comparing the (a) measured lesion diameters for the plate electrodes and (b) density score for each experimental frequency. Box plots (red) which share a common symbol (+, *, or #) were not statistically different from each other for (a) *α *= 0.1 and (b) *α *= 0.05. The average value and standard deviations are represented by green and blue lines respectively. The box plots represent the interquartile range between the 25^th ^and 75^th ^data percentiles. The largest lesions developed and the lowest density score was observed when pulses were applied at a frequency of 1 Hz

The average applied voltage to distance ratio between the plates for the frequency trials was 962 V/cm, corresponding to an average applied voltage and tissue thickness of 696.9 +/- 141.7 V and 7.3 +/- 1.5 mm, respectively. Lesions from these trials developed over 22 hours post-treatment, and were 2.5 cm in diameter on average (125% electrode diameter); with a minimum lesion of 2 cm occurring at 0.25 Hz and 936 V/cm, and maximum lesion of 3.2 cm occurring at 1.0 Hz and 950 V/cm. Though not dramatically significant, the results suggest that lesion sizes were on average greatest at 1 Hz and decreased as the frequency increased or decreased. The lesions which developed after treatments applied at 0.25 and 4 Hz were statistically smaller (*α *= 0.1) than those which developed for treatments applied at 1 Hz (Figure 3). Future studies will investigate the role of pulse parameters such as repetition rate, duration, magnitude and number on lesion volume.

Analysis of the treated tissue reveals a uniform treatment region that extended cylindrically through the tissue with no visible damage distal to the treatment regions. This resulted in calculated treated volumes between 1.97 cm^3 ^and 6.37 cm^3 ^for corresponding tissue thicknesses of 0.628 and 0.792 cm.

**Figure 4:**
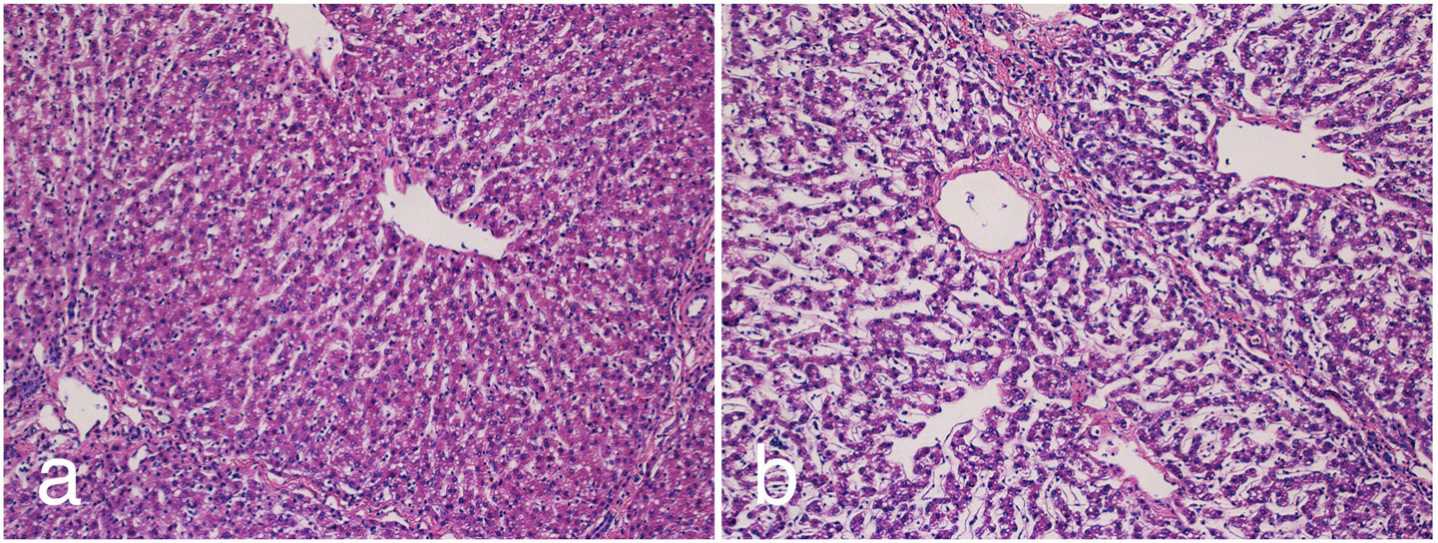
**Histological comparison of untreated liver tissue to areas which have undergone mild IRE treatments showing preservation of connective tissue and blood vessels**. Histological comparison of untreated liver tissue to areas which have undergone mild IRE treatments showing preservation of connective tissue and blood vessels. Samples stained with H&E from (a) untreated and (b) ninety nine, 100 μs, 1000 V/cm pulses using plate electrodes 24 hours of cardio emulation perfusion at 10×.

Histological examination 24 hours post-treatment indicates that treated regions exhibit cell death (Figure 4b) compared to controls (Figure 4a). Hepatic acini in pigs are bordered by connective tissue, which contains blood vessels and biliary structures, and have a prominent cord architecture terminating in a hepatic venule. In areas adjacent to energy delivery, hepatic cell cords were well preserved, with mildly vacuolated hepatocytes (an expected finding at 24-hour *ex vivo *machine perfusion cycle). Sinusoidal structure in untreated areas is open, reflecting the flow of perfusate between hepatic artery/portal vein and hepatic vein. N-TIRE treatment disrupts hepatic cords and induces cell degeneration (Figure 4b). Preservation of major acinar features, including connective tissue borders and blood vessels, is evident. In zones of N-TIRE treatment, cell cords were indistinct and membranes lining sinusoids are fragmented to varying degrees.

**Figure 5:**
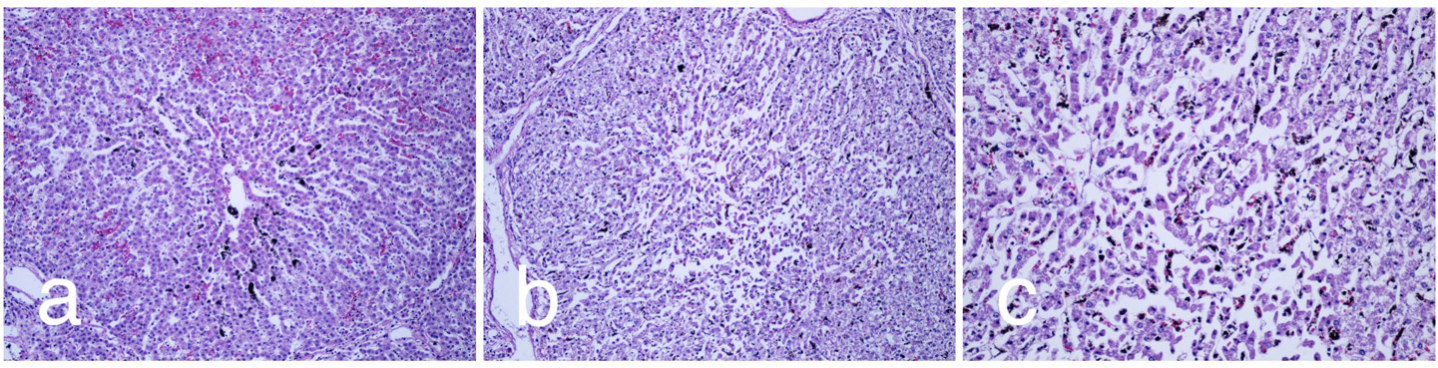
**IRE treatments result in hepatocytic cord disruption and cell delamination**. (a) A section of untreated liver after 24 hours of perfusion. Sections of the same liver treated with 90, 1500 V/cm, 100 μs pulses at 4 Hz using needle electrodes after 24 hours of perfusion at (b) 10× and (c) 20× magnification.

Pigs, like humans, have substantial septation of liver acini by thin bands of fibrous connective tissue that run between portal triads. This macrostructure had an effect on the distribution of lesions induced by electroporation. Lesions are confined within structural acini in a manner that at the edges of the electroporation field acini with lesions could border normal or nearly normal acini. Thus, the bands of connective tissue act as insulation for the electrical pulsing, an important observation when considering procedures for treating focal liver lesions with electroporation or for evolving an intact connective tissue/duct/vascular matrix for subsequent tissue engineering. Figure 5a shows a portion of untreated porcine liver with normal sinusoidal cell cords arrayed from portal tracts to central vein. Cell morphology is well preserved. Some vascular congestion with red blood cells is noted and there is also mild centrilobular biliary stasis. Mildly damaged porcine acini are observed in regions subjected to electroporation from needle electrodes (Figure 5b). The center of the acinus shows disruption of cord architecture and some cell degeneration and clumping. A higher magnification view of this area is shown in Figure 5c, where cellular changes are more readily appreciated. These treated regions display mild lesions consisting of hepatocytic cord disruption and cells delaminating from cord basal laminae. Mild biliary stasis is noted (dark pigment).

Administration of N-TIRE treatment, either with needle electrodes or with plate electrodes produced lesions in some hepatic acini that are distinctive. The severity of lesions within individual acini ranges from mild to moderately severe. Mild lesions consisted of small clumps of hepatocytes that detach from basal membranes. These cells show a loss of organization of fine intracellular structure and clumping of cytoplasm/organelles (Figure 5b-c).

**Figure 6:**
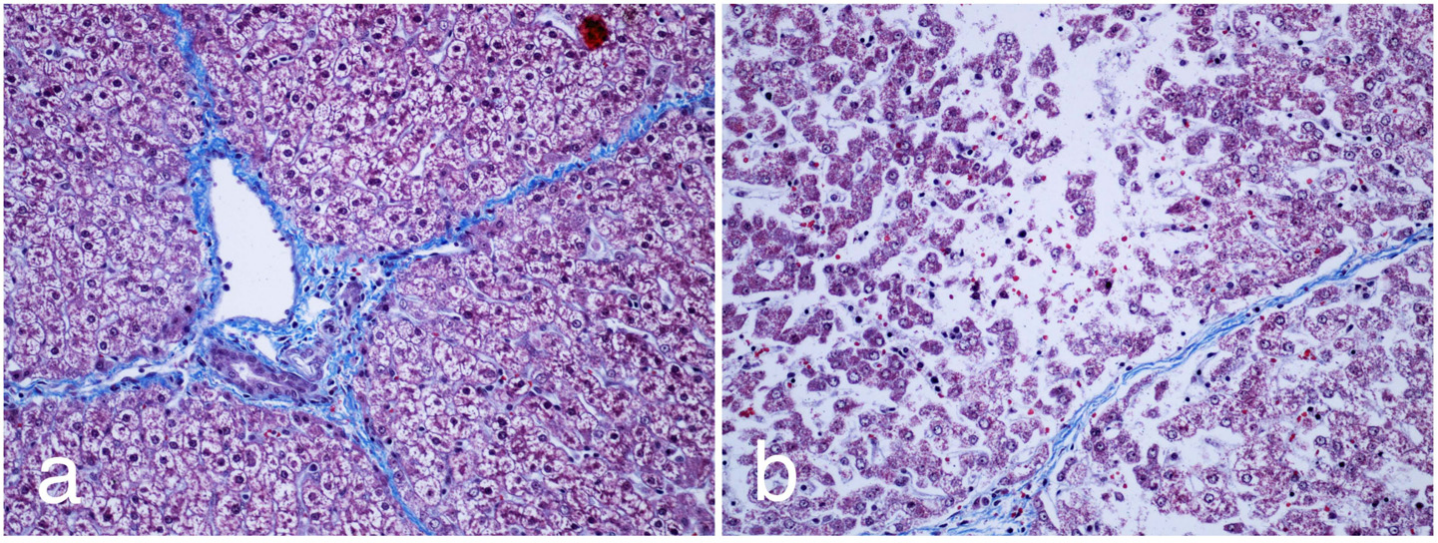
**Moderately severe lesions maintain bile ducts and vascular structures**. (a) A section of untreated liver after 24 hours of perfusion. (b) The same liver treated with 100, 1500 V/cm, 100 μs pulses at 1 Hz using needle electrodes after 24 hours of perfusion at 20× magnification.

Moderately severe lesions are readily discerned (Figure 6). Cells affected by the N-TIRE procedure show varying degrees of cell swelling and karyolysis (Figure 6b). Within individual acini, most cells are affected. In some acini, frank nuclear pyknosis and cellular degeneration is seen, with small clumps of hyperchromic cells unattached from basal membranes. In some acini, centrilobular biliary stasis is noted, with aggregation of bile pigments in distal sinusoidal spaces. In all cases, as noted, bridging bands of connective tissue, with intact bile ducts and vascular structures are seen, even immediately bordering acini with significant N-TIRE-induced tissue damage.

The density score for control samples was 0.87 +/- 0.0097 corresponding to approximately 87% of the histological tissue containing cells and extracellular material. Each treatment group had a statistically significant different density score versus the control (*α *= 0.01). The lowest density score of 0.509 +/- 0.069, was obtained for N-TIRE treatments where pulses were applied at a rate of 1.0 Hz. Additionally, treatments applied at 1.0 Hz resulted in a statistically significant lower density score as compared to all other treatment groups (α = 0.05).

## Discussion

To the best of our knowledge, this is the first work reporting the effect of non-thermal irreversible electroporation in an actively perfused organ. This effort is the first step towards creating decellularized tissue scaffolds that could be used for organ transplantation. This paper is aimed as a proof of concept to show that the cells may be removed, and therefore we targeted our study towards treating centimeter-scale regions of tissue. However, because N-TIRE procedures are dependent on the electric field to which a region of tissue is exposed, and thermal effects are mitigated by brief pulses with intervals between pulses, it is possible to scale up N-TIRE procedures to treat larger regions of tissue and organs.

The clearance of cellular debris was analyzed in this study using an image analysis algorithm as a preliminary method to determine the effectiveness of this technique. A more comprehensive study will include staining for primary and secondary antibodies, apoptotic markers, and DNA [16] and analysis of these samples via electron microscopy. Assays to determine the quantity of sulfated glycosaminoglycans, elastin, and collagen will be used as a measure of success of this method to preserve the important proteins in the extracellular matrix [13]. Additionally, biodegradation evaluation [13] and analysis of the vascular structure [14] must be completed before cell seeding and animal studies can be conducted.

The results reported here were localized to volumes of tissue up to 6.37 cm^3 ^for a single N-TIRE treatment. This can readily be expanded into much larger volumes by performing multiple treatments with the goal of creating decellularized structures for partial and full liver transplants. Analysis of the pulse repetition rate shows that the largest treated area and the lowest density score was achieved for a pulse frequency of 1 Hz. After 24 hours of perfusion, a maximum density score reduction of 58.5 percent had been achieved and cellular debris remained within the tissue construct. Since cell viability in the treatment regions was minimal, this is likely due to the combination of three factors; adhesion of cellular debris to the extracellular matrix, low physiological flow rates and pressures at the lobule level, and possible damage to the microvasculature by the N-TIRE treatments.

Although electroporation has been shown to preserve major blood vessels, vascular occlusion after electroporation has been reported in the literature under multiple treatment regiments including work done by Edd *et al. *[51] (a single 20 ms, 1000 V/cm pulse), Sersa *et al. *[52] (eight, 100 *μ*s, 1300 V/cm pulses at 1 Hz) and Nuccitelli *et al. *[53] (three hundred, 300 ns, 40 kV/cm pulses at 0.5 Hz) and is reportedly due to two mechanisms. The first is a rapid onset of temporary vasoconstriction due to reflex vasoconstriction of vascular endothelial cells lasting between 1 and 3 minutes [54]. The second, slower mechanism is due to the disruption of the microfilament and microtubule cytoskeletal networks which are necessary for maintaining cell function and structure [55]. This decrease in blood flow has been observed lasting up to 4 to 8 hours after electroporation of *in-vivo *tumors [56] before partially recovering to normal physiological values after 24 hours [57,58]. Thus, electroporation induces profound but essentially transient and reversible decline in blood flow [56]. This phenomenon may have occurred during the course of perfusion *ex-vivo*, though it was not directly observed, and it may have an effect on the clearance of cellular debris from the vascular network.

Additionally, the branching network of vessels within the liver produces a system with low pressures and fluid velocities at the capillary level and within individual lobules. The combination of physiological geometry and the loss of fine capillary structure caused by the N-TIRE treatments may have resulted in local sheer stresses which were not significant enough to fully clear cellular debris from the tissue. Removal of this debris is essential in minimizing immune response of recipients. Future work will focus on optimizing treatment and perfusion protocols to minimize disruption of the microvasculature network while enhancing the clearance of debris. This may include the continuous application of sub 1000 V/cm pulses at 1 Hz and perfusion at higher than physiological pressures which we believe will enhance the decellularization process. Recently developed chemical decellularization processes require perfusion cycles of up to 72 hours [14] for the complete removal of cellular material from a rat liver matrix and extension of N-TIRE treatment and perfusion cycles to these durations may be necessary to achieve complete decellularization.

Both external plate electrodes and needles placed within the tissue produced clearly delineated regions of cell death. Plate electrodes produced circular surface lesions, which when appeared cylindrical in shape in sectioned samples and extended between the top and bottom electrodes. Sections of tissue treated with needle electrodes produced oval shaped surface lesions which extended through the tissue.

Needle punctures damaged the tissue structure and provided an alternative path for fluid to flow. Rather than returning through the vasculature, some perfusate escaped the organ through the punctures hindering the perfusion process. Due to this, treatment of an entire organ using needle electrodes is likely not possible and external electrodes appear to be the best method of inducing N-TIRE in large tissue volumes.

In N-TIRE areas, cell death was directly related to energy delivered. Close to electrode placements, over 90% of the cells were degenerate and in varying stages of lysis. In the more reversibly energized zone, cell disruption was 20-30% of cells. Other cells may have been degenerate or leaky, but not morphologically abnormal. We have observed that the machine perfusion system can mobilize large amounts of cellular debris, a significant benefit for tissue engineering.

In addition to producing decellularized tissue scaffolds, this method provides an ideal platform to study the effects of pulse parameters such as pulse length, repetition rate, and field strength on whole organ structures. Additionally, since we have direct control over the electrical properties of the perfusate, this could serve as a model for examining the effects of N-TIRE on diseased or cancerous organs with unique electrical or physical properties.

The development of engineered materials to replicate the structure and function of thoracic and abdominal organs has achieved only limited success. Large volumes of poorly-organized cells and tissues cannot be implanted due to the initial limited diffusion of oxygen, nutrients and waste [59,60]. Despite this, researchers have made some progress toward complete organ regeneration. For instance, mouse renal cells, grown on decellularized collagen matrices and implanted into athymic mice, developed nephron-like structures after 8 weeks [9]. In addition, five millimeter thick porous polyvinyl-alcohol (PVA) constructs, implanted in mice and then injected with hepatocytes, developed liver-like morphology over the course of one year [7]. However, cell survival and proliferation in each of these structures was limited to a few millimeters from a nutrient source [7].

The resulting scaffolds from N-TIRE plus perfusion maintain the vasculature necessary for perfusion into structures far beyond the nutrient diffusion limit that exists for non-vascularized structures. Since the temperature of the perfusate used can be as low as 4°C, thermal aspects associated with Joule heating are negligible. This provides an ideal platform in which to explore the effects on the cells and tissue of electric fields in isolation from the effects of thermal damage. Additionally, the low temperature of the organ compared to *in vivo *applications may allow for the application of much higher voltages to attain appropriate electric fields for decellularizing thicker structures without inducing thermal damage. This is important since the thickness of a human liver can exceed 10 cm in some regions.

When planning to decellularize tissues and organs undergoing active perfusion, the treatment region of decellularized tissue may be predicted through numerical modeling. From the lesion sizes and numerical model used here, when decellularizing an entire organ for a transplantable scaffold, the protocol should expose all of the tissue to an electric field of 423 +/- 147 V/cm. This will ensure complete cell death, allowing comprehensive reseeding of the scaffold with the desired cells, thus minimizing the effects of recipient rejection. The threshold found here is slightly lower than the approximate 500-700 V/cm values reported in previous investigations [34,35]. This may be a result of the unique pulse parameters used (e.g., pulse number) or an inherent increased sensitivity of the cells to the pulses when under perfusion. The variability in the electric field threshold may result from the multiple inhomogeneous characteristics of the tissue anatomy and structure, such as the vascular system and tissue thickness, leading to lesions that were not perfectly circular.

The continuous active machine perfusion methods utilized here in the decellularization process may also be advantageous for recellularization. Once the decellularization process is complete, it should be possible to reseed the scaffold without risking damage attendant with removing the newly-created scaffold from the perfusion system. Since the arterial and venous supplies are individually addressable, multiple cell types can be delivered simultaneously to different regions of the organ. Similarly, retrograde perfusion through the biliary system may be the ideal pathway in which to deliver hepatocytes for the reseeding process.

Lesions seen microscopically are clearly indicative of a mechanism and morphology for cellular stripping using electroporation. It is very interesting that even at 24 hours, when using the N-TIRE parameters described here, there is only a modest loss of acinar architecture. More stringent conditions of energy delivery could likely alter this, but this might induce damage to important connective tissue and vascular structures. Addition of adjuvant cytotoxic agents, enzymes, and detergents in the perfusion fluid also might modulate the severity and temporal nature of cell stripping. Logically, it is much better to build on mild conditions, preserving important architecture for tissue engineering purposes, than to rapidly obliterate cells and stroma. The ability to manage the period of perfusion and conditions of perfusion with the cardioemulation system has clear advantages for this gradual, evolutionary approach to decellularization and eventual recellularization of liver.

## Conclusions

This study investigated the ability to develop decellularized tissue scaffolds using N-TIRE on organs undergoing active perfusion. Porcine livers were harvested and placed under active mechanical perfusion while N-TIRE electrical pulses were applied using plate and needle electrodes. Livers were removed from the perfusion system and the resultant lesions and control regions were examined histologically and a density score improvement of 58.5 percent was observed. Through numerical modeling of the electric field distribution from the pulse applications, it was found that an N-TIRE threshold of 423 +/- 147 V/cm may be used to predict the affected area. The continuous active machine perfusion method utilized during the decellularization process in this study provides the necessary platform for scaffold recellurization, a vital aspect required for practical organ transplantation techniques.

## Competing interests

Davalos has a patent pending on this technology.

## Authors' contributions

RVD and JR conceived and designed the experiments. MBS, REN, and PAG performed and analyzed the experiments and numerical modeling. DG supplied critically important intellectual content in revising the manuscript. All authors read and approved the final manuscript.
